# Disparities in Cervical and Breast Cancer Screening Among Sexual Minority Women in Japan: A Comparative Cross-Sectional Study

**DOI:** 10.3390/cancers17091411

**Published:** 2025-04-23

**Authors:** Akemi Hara, Akihiko Ozaki, Michio Murakami, Hiroaki Saito, Mika Nashimoto, Daisuke Hori, Masaharu Tsubokura, Kenji Gonda, Masahiro Wada, Kazunoshin Tachibana, Tohru Ohtake, Takahiro Tabuchi

**Affiliations:** 1 Medical Governance Research Institute, Minato City 108-0074, Japan; 2119089@alumni.tus.ac.jp; 2 Center for Infectious Disease Education and Research, The University of Osaka, Suita City 565-0871, Japan; michio@cider.osaka-u.ac.jp; 3 Soma Central Hospital, Fukushima Medical University School of Medicine, Fukushima City 960-1295, Japan; hiros@fmu.ac.jp; 4 Kameda Medical Center Breast Center, Tokyo 173-8605, Japan; 24km20010pr@stu.teikyo-u.ac.jp; 5 Institute of Medicine, University of Tsukuba, Tsukuba City 305-8575, Japan; daisuke_hori@md.tsukuba.ac.jp; 6 Department of Radiation Health Management, Fukushima Medical University School of Medicine, Fukushima City 960-1295, Japan; tsubo-m@fmu.ac.jp; 7 Jyoban Hospital of Tokiwa Foundation, Fukushima Medical University, Fukushima City 960-1295, Japan; gondake@kind.ocn.ne.jp; 8 Jyoban Hospital of Tokiwa Foundation, Utsunomiya Central Clinic, Utsunomiya City 320-0806, Japan; rope@helen.ocn.ne.jp; 9 Department of Breast Surgery, Fukushima Medical University, Fukushima City 960-1295, Japan; bakazu@fmu.ac.jp (K.T.); trcyn@cc.fmu.ac.jp (T.O.); 10 Division of Epidemiology, School of Public Health, Tohoku University Graduate School of Medicine, Sendai City 980-8575, Japan; takahiro.tabuchi.a5@tohoku.ac.jp

**Keywords:** cervical cancer screening, breast cancer screening, sexual minorities, healthcare disparities, Japan

## Abstract

**Objectives**: While health disparities affecting sexual minority women are well-documented globally, little is known about cancer screening behaviors among sexual minority women in Japan. Following our previous study on breast cancer screening, this study examined cervical cancer screening participation patterns and compared screening behaviors between both cancer types among sexual minority women in Japan. **Methods**: We analyzed data from 13,730 individuals with female sex assigned at birth who participated in a nationwide online survey between September and November 2023. Multinomial logistic regression was used to examine factors associated with screening participation, comparing sexual minority women (n = 2685) and women who are not part of a sexual minority (n = 11,045). Among participants aged 40 and above (n = 8933), we compared participation patterns between cervical and breast cancer screenings. **Results**: Sexual minority women showed significantly lower cervical cancer screening rates compared to women who are not part of a sexual minority (38.7% vs. 45.6%, *p* < 0.001), with a wider disparity than observed in breast cancer screening (43.4% vs. 45.9%, *p* < 0.001). Among those aged 40 and above, sexual minority women were more likely to skip both screenings (35.0% vs. 27.2%) and less likely to participate in both (55.0% vs. 62.6%). Additionally, our analysis revealed that participants with a current mental disorder (i.e., those reporting ongoing mental health issues) were more likely to intend to undergo cervical cancer screening (aOR = 1.39, 95% CI = 1.15–1.67, *p* = 0.001). In contrast, among bisexual participants and those classified as having “other” mental health conditions—defined as a history of mental health issues without current symptoms—exhibited significantly lower odds of being screened (aOR = 0.31, 95% CI = 0.11–0.82, *p* = 0.02). **Conclusions**: Significant disparities exist in cancer screening participation among sexual minority women in Japan, with more pronounced differences in cervical cancer screening compared to breast cancer screening. These findings highlight the need for targeted interventions addressing the unique barriers to gynecological care among sexual minority women.

## Simple Summary

This study addresses the important issue of lower participation in cervical and breast cancer screenings among sexual minority women (such as lesbian, bisexual, queer, and transgender individuals assigned female at birth) in Japan. The research aimed to identify differences in screening rates between sexual minority women and women who are not part of a sexual minority, and to understand the reasons behind these disparities. Using data from an extensive online survey involving over 13,000 participants, the study found that sexual minority women participate less frequently in cervical cancer screenings compared to other women, with significant gaps also observed in breast cancer screenings. Factors like marital status, insurance coverage, income, and mental health influenced these screening behaviors. These findings are valuable as they highlight the need for targeted healthcare strategies to improve cancer screening rates among sexual minority women, ultimately aiming to reduce healthcare inequalities and enhance preventive healthcare in Japan.

## 1. Introduction

Cancer screening remains a cornerstone of cancer prevention and public health, significantly reducing morbidity and mortality through early detection and timely treatment [1,2]. However, global evidence reveals that individuals often resist screening for distinct reasons depending on the cancer type. Cervical cancer screening is commonly avoided due to discomfort with pelvic examinations and cultural taboos related to gynecological care [3], while resistance to breast cancer screening tends to stem from fear of pain and underestimation of personal susceptibility [4]. These psychological and cultural deterrents are particularly pronounced among sexual minority women (including lesbian, bisexual, and queer individuals, as well as transgender people assigned female at birth), who face additional structural and social barriers to accessing healthcare. These include experiences of stigma and discrimination, lack of inclusive clinical environments, and misconceptions—both within the general public and among medical professionals—regarding their cancer risk profiles [5,6].

In Japan, the situation is further complicated by nationally low participation rates in both cervical and breast cancer screening compared to many Western countries [7]. While the introduction of mobile mammography has modestly expanded access, screening uptake remains uneven, especially among vulnerable groups such as the uninsured [7,8]. Cervical cancer screening presents additional obstacles due to the procedure’s invasive nature and persistent stigma surrounding gynecological health. These challenges were exacerbated by the Japanese government’s 2013 suspension of proactive HPV vaccine promotion, which was only reversed in 2022 [9,10]. As a result, more weight has been placed on screening as the primary mode of cervical cancer prevention. Against this backdrop, our previous work demonstrated that sexual minority women in Japan have significantly lower rates of breast cancer screening compared to women who are not part of a sexual minority, even after controlling for socioeconomic status [11]. This finding aligns with international research, which has consistently shown that insurance status, marital status, and income levels are important determinants of cancer screening among marginalized populations [6,11].

Emerging evidence also points to the role of psychological well-being in influencing preventive health behaviors. Mental health issues such as depression, anxiety, and medical avoidance have been associated with reduced screening rates in the general population [12,13]. However, the extent to which mental health affects screening behavior among sexual minority women remains understudied, particularly in relation to cervical cancer screening, which may trigger more intense psychological discomfort due to its intimate nature.

Building on our prior study of breast cancer screening disparities, the present research expands the scope of inquiry to include cervical cancer screening among sexual minority women in Japan. We aim to evaluate whether sociodemographic factors previously identified—namely, insurance coverage, marital status, and annual household income—are also significantly associated with cervical cancer screening behavior. In addition, we newly examine the influence of mental health status, hypothesizing that psychological conditions may serve either as a barrier or motivator, depending on the type and timing of symptoms. Finally, by directly comparing predictors of breast and cervical cancer screening, we seek to identify both the shared and unique determinants of participation.

## 2. Materials and Methods

### 2.1. Data Collection

The survey was conducted via an online platform and comprised both a follow-up survey and a new survey using a unified questionnaire.

- Survey Timeline: The survey period spanned from 25 September to 17 November 2023.
- Follow-Up Survey: Among the respondents from studies conducted in 2015–2023 (JASTIS + JACSIS), 46,840 individuals who were still contactable by the survey company were invited to participate. The follow-up survey was conducted between 25 September and 17 November 2023, yielding 26,872 responses, which corresponds to a response rate of 57.37%.
- New Survey: In addition to the follow-up survey, a new survey was administered to panel members aged 16–79 using the same questionnaire. This survey was conducted from 7 November to 17 November 2023 and generated 6128 responses. Combined with the follow-up survey responses, the total number of respondents reached 33,000.
- Recruitment and Administration: Survey invitations were disseminated by a research agency employing simple random sampling, stratified by sex, age, and prefecture. Participants provided web-based informed consent prior to completing the survey online. They had the option to skip questions or discontinue the survey at any point. The survey was automatically closed once the target quotas for each demographic category were met.
- Ethical Considerations and Incentives: All procedures adhered to the ethical standards outlined in the Helsinki Declaration (1975, revised 2013) and were approved by the Research Ethics Committee of the Osaka International Cancer Institute (approval number 20084, 19 June 2020). The study complied with Japan’s Act on the Protection of Personal Information. Participants received “Epoints” as an incentive, which could be redeemed for internet shopping or converted to cash.

### 2.2. Settings and Participants

From the total participants (33,000), we excluded invalid responses (4519), individuals with male sex assigned at birth (14,071), and individuals with female sex assigned at birth who did not answer cervical cancer screening questions (680), resulting in 13,730 valid responses from individuals with female sex assigned at birth. Participants were recruited through Rakuten Insight (Tokyo, Japan), targeting individuals aged 16–79. Recruitment began on 25 September 2023, and ended on 17 November 2023, once the target sample size was reached. The data comprised 26,872 responses from follow-up surveys (response rate: 57.37%) and 6128 from new surveys conducted between 7 and 17 November 2023. To ensure representativeness, participants were selected to align with Japan’s demographic distribution in terms of age, sex, and prefectural residence. Web-based informed consent was obtained prior to participation, ensuring ethical procedures.

Although the dataset of 33,000 responses offers a robust foundation for analysis, the reliance on an internet-based survey introduces limitations. Populations with restricted access to digital tools or lower proficiency in using the internet—such as elderly individuals or those from socioeconomically disadvantaged groups—may be underrepresented. Consequently, there is a need for careful consideration when extrapolating these findings to the wider Japanese population due to the potential for selection bias.

### 2.3. Outcome Variables

Participants were asked whether they had undergone breast cancer screening (e.g., mammography or breast ultrasound) and cervical cancer screening (e.g., cervical cytology) within the past two years. For analysis, screening status was classified into three groups. “Screened” included all participants who had undergone screening, regardless of their results. “Attempted but not screened” referred to those who intended to undergo screening but had not yet completed it, reflecting an intention but an inability to complete the screening process. “Not screened” included participants who had neither undergone screening nor planned to do so. For those in the “screened” category, screening outcomes were further classified as “No abnormality” for those with no abnormal findings, “Abnormality” for those who received abnormal results, and “Unknown result” for those who were unaware of or had not yet received their results.

To ensure transparency, the exact survey question and response options used in this study are provided in the Supplementary Materials to clarify how screening status was assessed and categorized in the analysis.

### 2.4. Exposure Variables

This study used the same variables as our previous work on breast cancer screening [11], with the addition of mental health status. To confirm their relevance, univariable multinomial logistic regressions were performed for each variable using likelihood ratio tests. All variables showed significant associations with the outcome (*p* < 0.05), as shown in Supplementary Table S1.

Key variables influencing screening behaviors were analyzed with defined reference categories. Insurance enrollment status was categorized into “National Health Insurance” (reference), “Employee’s Health Insurance” (which includes all types of employee health insurance), “Uninsured”, and “Other” (which includes public assistance and various alternative insurance or financial support systems). Marital status was categorized into “unmarried” (reference) and “married”, with the latter including legally married individuals, common-law partnerships, and same-sex partners living as a married couple, regardless of official marriage registration. Annual household income was stratified into “less than JPY 5 million” (reference), “JPY 5–10 million”, “JPY 10 million or more”, and “unknown.” Alcohol consumption, smoking status, and mental health status were categorized into categorical variables for analysis. For alcohol consumption, participants were classified as a “Drinker” (including those who drink occasionally or almost daily) or “Non-drinker” (including those who have never consumed alcohol, tried it once but do not use it regularly, or used to drink but have stopped). For smoking status, participants were categorized as a “Smoker” (including those who smoke occasionally or almost daily) or “Non-smoker” (including those who have never smoked, tried smoking but do not smoke regularly, or those who used to smoke but have quit). For mental health status, we categorized participants into three groups to ensure a broader assessment of mental health conditions not limited to depression but also including other psychiatric disorders. Participants who reported never having had any mental disorder were classified as “No mental disorder”. Those who had a past mental disorder but were not currently diagnosed were categorized as “Other”. Participants who were currently diagnosed with a mental disorder, regardless of treatment status, were classified as “Yes”. In each case, “Non-drinker”, “Non-smoker”, and “No mental disorder” were used as the reference categories in the analysis. Age was grouped into 10-year increments, starting at “20–29 years” (reference). These references were used as baselines in multinomial logistic regression to examine disparities in screening participation.

### 2.5. Data Analysis

In Japan, cervical cancer screening (or the pap smear) is recommended biennially from age 20, and breast cancer screening (mammography) biennially from age 40 [12,13]. Both are offered through municipal programs and private clinics, with local government implementation. Screening rates remain low—43% for cervical and 45% for breast cancer—compared to other developed nations. These rates are significantly lower than the 70% participation rates seen in many Western countries [14,15,16].

To better understand the intentions and barriers to screening for future interventions, this study at first included participants under 40 for both cervical and breast cancer screenings. Independent variables (e.g., cervical cancer screening status, health insurance, and income) were analyzed between sexual minority women and women who are not part of a sexual minority using descriptive statistics and chi-square tests. Multivariable multinomial logistic regression was conducted with the “non-screened group” as the reference, comparing it to the “screened” and “intending to screen” groups. Stepwise variable selection was used, and ORs with 95% CIs were calculated. Variance inflation factors (VIFs) were calculated to assess multicollinearity, and results indicated that age had a high VIF, suggesting the presence of multicollinearity. Consequently, age was excluded from the final model to ensure the robustness of the analysis.

Subsequently, we filtered the dataset to align with the inclusion criteria of the breast cancer screening data. A total of 11,056 participants who provided responses for both breast and cervical cancer screenings were selected. For contingency table analysis, we further restricted the dataset to participants aged 40 years and older to examine screening status by sexual minority women and women who are not part of a sexual minority. For the purpose of analysis, participants who expressed an intention to undergo screening were categorized as having completed screening. Screening proportions were then calculated for the overall sample, as well as separately for sexual minority women and women who are not part of a sexual minority. Chi-square tests were conducted to evaluate differences in screening distributions between sexual minority women and women who are not part of a sexual minority.

We then performed multinomial logistic regression analysis, using the covariates from the cervical cancer screening study. The analysis focused on participants aged 40 and above (8933) and explored the relationship between individual background characteristics and screening status, with participation in both screenings (5465) as the reference group. Comparisons were made among those who participated only in breast cancer screening (681), only in cervical cancer screening (226), or in neither screening (2561).

All data analyses were conducted using R version 4.2.1, based on statistically valid methods. As a sensitivity analysis, to robustly evaluate the identity of sexual minority women, we have provided a detailed breakdown of demographic characteristics and screening behaviors within sexual minority women subgroups in Supplementary Table S2. As shown in Supplementary Tables S3 and S4, we performed analyses excluding or recategorizing respondents with unclear sexual orientation, as previously utilized in our breast cancer screening study. As shown in Supplementary Table S5, we conducted weighted regression analyses, setting the proportion of sexual minority women individuals to 9.7%—a figure derived from the Dentsu “sexual minority women+ Survey 2023”—to assess the robustness of our findings. Finally, building on the above, we further expands on our analysis by showing multinomial logistic regressions performed for each subcategory of sexual minority women (with the exception of lesbian women due to their small sample size) to explore differences in their background characteristics.

### 2.6. Ethics Statements

This study received ethical approval from the Osaka International Cancer Institute. The initial approval, under the study title “Evaluation of Social and Health Disparities Caused by the COVID-19 Pandemic in Japan”, was granted on 19 June 2020 (approval number: 20084), prior to the implementation of the survey. The current research, an extension of the original study, received additional approval (approval number: 20084-12) on 28 March 2024 and subsequently from the Tohoku University Graduate School of Medicine Ethics Committee (approval numbers: 2024-1-231 on 27 June 2024, and 2024-1-517 on 22 October 2024), in accordance with the 2020 ethical guidelines prior to the initiation of survey activities.

## 3. Results

Table 1 presents the descriptive statistics. The cervical cancer screening rate was lower in the sexual minority women compared to the women who are not part of a sexual minority (38.7% vs. 45.6%, *p* < 0.001). A higher proportion of sexual minority women were in their 20s (24.7% vs. 18.2%, *p* < 0.001). The distribution of other covariates was generally similar to those observed in breast cancer screening data, with the exception of mental health status, which was notably higher in the sexual minority women (9.1% vs. 5.6%, *p* < 0.001), highlighting its significance as a new variable in this study.

VIFs were calculated to assess multicollinearity. Age had a high VIF of 31.37, indicating multicollinearity, and was excluded from the final model. All other variables had VIF values below 10, with household income showing the highest value of 7.06, indicating acceptable levels of independence among variables.

Supplementary Table S2 presents a detailed breakdown of demographic characteristics and screening behaviors among sexual minority women subgroups and women who are not part of a sexual minority. Notably, bisexual participants reported the highest rates of mental health issues (19.5%), atypical screening results (6.6%), and the largest proportion of women in their 20s (51.1%), while homosexual participants reported a rate of 13.3% for mental health issues. In contrast, homosexual participants had the lowest cervical cancer screening rate (31.1%), compared with an overall rate of 38.7%.

Table 2 presents the results of the multivariable multinomial logistic regression analysis, which align with those from our previous breast cancer study, where most findings remained statistically significant. After adjusting for confounders, sexual minority women showed significantly lower odds of both cervical cancer screening (adjusted OR 0.75, 95% CI 0.68–0.83, *p* < 0.001) and screening intention (aOR 0.63, 95% CI 0.56–0.71, *p* < 0.001) compared to women who are not part of a sexual minority. Notably, mental health status showed a significant positive association with screening behavior. Individuals with a history of mental health issues (aOR 1.18, 95% CI 1.04–1.34, *p* = 0.01) were more likely to participate in screening. Similarly, those with current mental health conditions were more likely to intend to undergo screening (aOR 1.39, 95% CI 1.15–1.67, *p* = 0.001).

The consistency of these results is further supported by the analyses presented in Supplementary Tables S3–S5, which confirm the robustness of the findings across different model specifications and sample definitions. Notably, Supplementary Table S3 (excluding “unsure” participants) and Supplementary Table S4 (treating “unsure” as a separate category) both show similar patterns of association, suggesting that our conclusions are not sensitive to how sexual minority subgroups are classified. In addition, the weighted multinomial logistic regression in Supplementary Table S5 demonstrates nearly identical significance levels, further reinforcing the reliability and generalizability of our findings.

Analyses stratified (Table 3) by sexual orientation (“bisexual”, “other”, “undecided”, and “unsure”) revealed several significant associations (*p* < 0.05). Being married increased the odds of having undergone cervical cancer screening in all subgroups (bisexual: aOR = 2.85, 95% CI = 1.23–6.57, *p* = 0.01; other: aOR = 1.83, 95% CI = 1.19–2.82, *p* = 0.006; undecided: aOR = 1.65, 95% CI = 1.17–2.34, *p* = 0.005; unsure: aOR = 1.94, 95% CI = 1.50–2.50, *p* < 0.001). Among bisexual participants, “other” mental health status was negatively associated with being screened (aOR = 0.31, 95% CI = 0.11–0.82, *p* = 0.02). In the “other” group, holding employee’s health insurance elevated the odds of both being screened (aOR = 2.07, 95% CI = 1.33–3.21, *p* = 0.001) and intending to screen (aOR = 2.33, 95% CI = 1.32–4.10, *p* = 0.003). For “undecided” individuals, being uninsured lowered the odds of being screened (aOR = 0.19, 95% CI = 0.04–0.88, *p* = 0.03), while an annual income of JPY five to ten million had a positive association (aOR = 1.98, 95% CI = 1.23–3.21, *p* = 0.005). Reporting “yes” for mental health in the “undecided” group was linked to higher odds of both being screened (aOR = 1.95, 95% CI = 1.09–3.51, *p* = 0.03) and intending to screen (aOR = 2.53, 95% CI = 1.32–4.85, *p* = 0.005). In the “unsure” subgroup, having employee’s health insurance was positively associated with intending to screen (aOR = 1.52, 95% CI = 1.05–2.20, *p* = 0.03), whereas “other” insurance reduced the odds of having been screened (aOR = 0.43, 95% CI = 0.25–0.74, *p* = 0.002).

Table 4 shows that several factors significantly influence cervical cancer screening participation among sexual minority women, aligning with findings for breast cancer screening. Participants with employee health insurance were more likely to undergo screening compared to those with national health insurance (aOR: 1.28, 95% CI: 1.07–1.53, *p* = 0.01), while uninsured individuals had significantly lower odds (aOR: 0.21, 95% CI: 0.08–0.55, *p* = 0.001). Married individuals were more likely to participate than unmarried ones (aOR: 1.84, 95% CI: 1.54–2.20, *p* < 0.001). However, for the intending to screen group, the association was weaker but still significant (aOR: 1.27, 95% CI: 1.01–1.59, *p* = 0.04). Additionally, an annual household income between JPY 5 and 10 million was positively associated with screening participation (aOR: 1.43, 95% CI: 1.12–1.84, *p* = 0.01), though no significant association was observed for screening intention (*p* = 0.55). Higher incomes (>JPY 10 million) were not significantly associated with either screening participation (*p* = 0.56) or intention (*p* = 0.46). Alcohol consumption was also associated with increased participation (aOR: 1.26, 95% CI: 1.05–1.52, *p* = 0.01) and screening intention (aOR: 1.30, 95% CI: 1.03–1.64, *p* = 0.03). Meanwhile, smoking and mental health status showed no significant associations with participation (*p* = 0.84 and *p* = 0.14, respectively). However, individuals with current mental health conditions were more likely to intend to screen (aOR: 1.55, 95% CI: 1.07–2.24, *p* = 0.02). These findings are consistent with factors influencing breast cancer screening participation, highlighting shared barriers and facilitators for both screenings.

Supplementary Table S6 illustrates that sexual minority women had a higher likelihood of skipping both cervical and breast cancer screenings (35.0% vs. 27.2%) and a lower likelihood of participating in both screenings (55.0% vs. 62.6%) compared to women who are not part of a sexual minority. The proportion of those who participated in cervical cancer screening but not breast cancer screening was slightly higher among sexual minority women (2.9% vs. 2.5%), while the proportion of those who participated in breast cancer screening but not cervical cancer screening was slightly lower (7.1% vs. 7.8%). A significant difference was observed between sexual minority women and the women who are not part of a sexual minority (*p* < 0.001).

Table 5 presents the results of multinomial logistic regression analyses comparing the odds of being in each screening participation category—breast cancer screening only, cervical cancer screening only, or non-participation—relative to complete screening, based on various demographic, behavioral, and mental health factors. Sexual minority women had higher odds of being non-participants in any screening compared to those who participated in all screenings (aOR 1.29, 95% CI 1.14–1.45, *p* < 0.001). However, there were no significant differences observed for participation in cervical cancer screening only (*p* = 0.22) or breast cancer screening only (*p* = 0.62) among sexual minority women. Participants with employee health insurance were less likely to participate only in breast cancer screening (aOR 0.45, 95% CI 0.38–0.54, *p* < 0.001) and only in cervical cancer screening (adjusted OR 0.70, 95% CI 0.53–0.92, *p* = 0.01), or to be non-participants (aOR 0.48, 95% CI 0.43–0.53, *p* < 0.001). Married individuals were less likely to participate only in breast cancer screening (aOR 0.78, 95% CI 0.65–0.92, *p* = 0.004) or to be non-participants (aOR 0.81, 95% CI 0.73–0.90, *p* < 0.001). Smokers were more unlikely to participate only in breast cancer screening (aOR 0.72, 95% CI 0.53–0.98, *p* = 0.03) but more likely to participate only in cervical cancer screening (aOR 1.58, 95% CI 1.08–2.32, *p* = 0.02).

Mental health status showed varying associations with screening participation. Individuals with current mental health conditions were significantly more likely to participate only in cervical cancer screening compared to those who participated in all screenings (aOR 1.70, 95% CI 1.06–2.74, *p* = 0.03). However, mental health status was not significantly associated with participation in breast cancer screening only (*p* = 0.16). Notably, individuals with ‘Other’ mental health status—defined as having a history of mental health issues without a current diagnosis—had significantly lower odds of being non-participants compared to those with no mental health history.

Higher household income was significantly associated not only with lower odds of being a non-participant (aOR 0.52, 95% CI 0.41–0.65, *p* < 0.001) but also with decreased odds of participating in breast cancer screening alone (aOR 0.52, 95% CI 0.35–0.78, *p* = 0.002). Alcohol consumption was not significantly associated with breast or cervical cancer screening alone but was linked to a lower likelihood of being a non-participant (aOR 0.87, 95% CI 0.79–0.96, *p* = 0.007). These findings highlight the role of socioeconomic and behavioral factors, including mental health status, in influencing screening behaviors across both cancer types.

## 4. Discussion

Our study reveals notable disparities in both breast and cervical cancer screening participation between sexual minority women and women who are not part of a sexual minority in Japan, with the gap being especially pronounced for cervical cancer screening.

One potential explanation for the greater disparity observed in cervical cancer screening is the inherent invasiveness of the procedure. Cervical examinations, which involve pelvic exams, can provoke heightened discomfort and anxiety. Cultural factors and past negative experiences with gynecological care may further exacerbate these concerns, particularly among sexual minority women who may already face challenges in accessing inclusive healthcare services [10,11]. These factors might contribute to a greater reluctance to participate in cervical cancer screening compared to breast cancer screening. Additional research is warranted to fully elucidate the mechanisms underlying these differences.

A key finding of our analysis is the significant role of marital status. Across our models, being married consistently increased the odds of undergoing screening. This suggests that factors associated with marriage, such as spousal support and household stability, may facilitate access to and engagement with preventive care. Our results align with previous research indicating that social support is a critical determinant of health service utilization.

Mental health emerged as a particularly complex factor in our analyses. In our overall sample, individuals reporting “other” mental health conditions tended to show higher adjusted odds of both having been screened and intending to screen. Similarly, those with a history of mental disorders were more likely to express an intention to undergo screening, although this was not reflected in significantly higher actual screening rates. These findings contrast with several previous studies—for example, those by Weinstein et al. (2015) and Yee et al. (2011)—which reported that mental health issues, particularly depression, often act as barriers to cancer screening due to reduced motivation and apprehension about healthcare encounters [17,18].

Our subgroup analyses, however, provide a more nuanced picture. Specifically, in the bisexual subgroup, reporting “other” mental health conditions was significantly associated with lower odds of being screened. This divergence underscores that the effect of mental health on screening behavior varies by subgroup, highlighting the importance of disaggregating non-heterosexual populations. Not all non-heterosexual groups experience mental health challenges in the same way, and our results suggest that, while the overall effect may appear positive, certain subgroups such as bisexual individuals may indeed face significant psychological barriers. In addition to mental health, other factors such as insurance type and income also played roles in screening behaviors, but the influence of marital status and mental health appeared particularly robust. Interventions must not assume a uniform impact of mental health challenges across all non-heterosexual groups but instead should be designed to address subgroup-specific barriers.

Collectively, these findings emphasize that a one-size-fits-all approach to increasing cancer screening uptake may not be effective for non-heterosexual populations. Instead, public health strategies should incorporate tailored interventions that account for the diverse experiences and needs within these groups. Future research should aim to recruit larger, more targeted samples that allow for more granular analyses of non-heterosexual subpopulations. This will help clarify the underlying mechanisms through which factors such as marital status and mental health influence screening behaviors and ultimately support the development of more effective, inclusive preventive care programs.

## 5. Implications

These findings highlight the need for targeted public health approaches to improve cancer screening rates among sexual minority women in Japan. Key strategies should include sexual minority women-specific health education, more inclusive healthcare environments, and addressing disparities in insurance coverage. Healthcare providers should be trained in sexual minority women’s health issues and cultural competency.

## 6. Limitations

This study’s cross-sectional design limits our ability to draw causal inferences, and our sample—recruited online from a general population—may not fully represent the broader community. Additionally, the lack of adjustment for education and other sociodemographic factors, together with the absence of detailed sexual behavior data, constrains our capacity to precisely assess HPV exposure risk.

Our use of broad, undefined categories such as “other”, “undecided”, and “unsure” (representing 18.2%, 27.2%, and 46.4% of respondents, respectively) may obscure important heterogeneity within sexual minority women, particularly regarding screening behaviors and associated risks via heterosexual contact. Moreover, because our survey instrument was not specifically designed to target sexual minority women populations, subgroup sizes—especially for lesbian women—are limited, and we did not explicitly distinguish transgender males (individuals with sex assigned at birth as female), leaving their representation uncertain.

Furthermore, mental health status, including depression, was assessed using self-reported data, which may be subject to reporting biases, personal perceptions, or differences in clinical diagnosis. This could influence the accuracy of mental health classification and its associations with cancer screening behaviors.

Lastly, our study found a higher proportion of sexual minority women (18.3%) compared to national surveys, likely due to the anonymity of the online survey format, suggesting that methodology can significantly influence self-disclosure and representation among sexual minority women.

These results provide hints for future research. For example, to better capture the unique barriers faced by diverse subpopulations, future studies should employ more targeted recruitment strategies, refine sexual orientation measures with clearer definitions, and incorporate comprehensive data on sexual behavior. In fact, even a single exploration of the distinction between self-identified gender and biological sex and their influence on screening behaviors may provide important insights into gender-specific healthcare access. Conducting international comparisons may also yield valuable insights into how differing health systems shape screening behaviors across various sociocultural contexts. Furthermore, incorporating in-person interviews could help address digital access limitations and provide a deeper understanding of the barriers and facilitators that influence cancer screening participation.

## 7. Conclusions

This study demonstrates significant disparities in cervical cancer screening participation among sexual minority women in Japan. Our findings indicate that, compared to their non-sexual minority counterparts, sexual minority women are less likely to participate in cervical cancer screening—an effect that is more pronounced than in breast cancer screening. These disparities appear to be influenced by a combination of factors, including access to inclusive healthcare services, socioeconomic determinants such as insurance type and marital status, and complex associations with mental health status.

In light of our findings, reducing cervical cancer screening disparities among sexual minority women requires targeted strategies including improved provider education, tailored outreach programs, and policy reforms that address their unique barriers to care—critical steps toward achieving health equity. Future research should further explore the nuanced interplay of socioeconomic and psychological factors across different subgroups to develop more effective, inclusive interventions.

## Figures and Tables

**Table 1:** Demographic characteristics of sexual minority women and women who are not part of a sexual minority (breast cancer status is based on previously reported data) [11].

	*Cervical Cancer Screening Status*					*Breast Cancer Screening Status*				
.	VIF	Total n (%)	Sexual Minority Women n (%)	Women Who Are Not Part of a Sexual Minority n (%)	Chi-Square Test	VIF	Total n (%)	Sexual Minority Women n (%)	Women Who Are Not Part of a Sexual Minority n (%)	Chi-Square Test
**Overall**	-	13,730 (100.0)	2685 (19.6)	11,045 (80.4)	-	-	11,056 (100.0)	2022 (18.3)	9034 (81.7)	-
**Age**	31.37				<0.001	29.93				<0.001
20s	-	2674 (19.5)	663 (24.7)	2011 (18.2)						
30s	-	2123 (15.5)	378 (14.1)	1745 (15.8)		-	2123 (19.2)	378 (18.7)	1745 (19.3)	
40s	-	2249 (16.4)	396 (14.7)	1853 (16.8)		-	2249 (20.3)	396 (19.6)	1853 (20.5)	
50s	-	2190 (16.0)	347 (12.9)	1843 (16.7)		-	2190 (19.8)	347 (17.2)	1843 (20.4)	
60s	-	2218 (16.2)	397 (14.8)	1821 (16.5)		-	2218 (20.1)	397 (19.6)	1821 (20.2)	
70s	-	2027 (14.8)	438 (16.3)	1589 (14.4)		-	2027 (18.3)	438 (21.7)	1589 (17.6)	
80s	-	249 (1.8)	66 (2.5)	183 (1.7)		-	249 (2.3)	66 (3.3)	183 (2.0)	
**Cancer Screening**	-	-	-	-	<0.001	-	-	-	-	<0.001
Screened	-	6079 (44.3)	1039 (38.7)	5040 (45.6)	-	-	5023 (45.4)	878 (43.4)	4145 (45.9)	-
No abnormality	-	5667 (93.2)	943 (90.8)	4724 (93.7)	<0.001	-	4637 (92.3)	813 (92.6)	3824 (92.3)	<0.001
Abnormality	-	242 (4.0)	52 (5.0)	190 (3.8)	-	-	247 (4.9)	38 (4.3)	209 (5.0)	-
Unknown result	-	170 (2.8)	44 (4.2)	126 (2.5)	-	-	139 (2.8)	27 (3.1)	112 (2.7)	-
Planned Screening	-	2768 (20.2)	443 (16.5)	2325 (21.1)	-	-	2450 (22.2)	331 (16.4)	2119 (23.5)	-
No Screening	-	4883 (35.6)	1203 (44.8)	3680 (33.3)	-	-	3583 (32.4)	813 (40.2)	2770 (30.7)	-
**Sexual minority women** **i** **dentity**	1.50	-	-	-	<0.001	1.53	-	-	-	<0.001
No	-	11,045 (80.4)	0 (0.0)	11,045 (100.0)	-	-	9034 (81.7)	0 (0.0)	9034 (100.0)	-
Yes	-	2685 (19.6)	2685 (100.0)	0 (0.0)	-	-	2022 (18.3)	2022 (100.0)	-	-
Lesbian	-	45 (1.7)	45 (1.7)	-	-	-	25 (1.2)	25 (1.2)	-	-
Bisexual	-	174 (6.5)	174 (6.5)	-	-	-	85 (4.2)	85 (4.2)	-	-
Other	-	489 (18.2)	489 (18.2)	-	-	-	428 (21.2)	428 (21.2)	-	-
Undecided	-	731 (27.2)	731 (27.2)	-	-	-	499 (24.7)	499 (24.7)	-	-
Unsure	-	1246 (46.4)	1246 (46.4)	-	-	-	985 (48.7)	985 (48.7)	-	-
**Insurance Status**	3.80	-	-	-	<0.001	4.49	-	-	-	<0.001
National Health Insurance	-	5898 (43.0)	1240 (46.2)	4658 (42.2)	-	-	4919 (44.5)	991 (49.0)	3928 (43.5)	-
Employee’s Health Insurance	-	7291 (53.1)	1232 (45.9)	6059 (54.9)	-	-	5817 (52.6)	916 (45.3)	4901 (54.3)	-
Other	-	429 (3.1)	172 (6.4)	257 (2.3)	-	-	118 (1.1)	38 (1.9)	80 (0.9)	-
Uninsured	-	112 (0.8)	41 (1.5)	71 (0.6)	-	-	202 (1.8)	77 (3.8)	125 (1.4)	-
**Marital Status**	3.40	-	-	-	<0.001	4.18	-	-	-	<0.001
Unmarried	-	7965 (58.0)	1329 (49.5)	6636 (60.1)	-	-	7243 (65.5)	1190 (58.9)	6053 (67.0)	-
Married	-	5765 (42.0)	1356 (50.5)	4409 (39.9)	-	-	3813 (34.5)	832 (41.1)	2981 (33.0)	-
**Annual Household Income (JPY)**	7.06	-	-	-	<0.001	8.38	-	-	-	<0.001
<5 million	-	5390 (39.3)	1089 (40.6)	4301 (38.9)	-	-	4384 (39.7)	835 (41.3)	3549 (39.3)	-
5–10 million	-	3584 (26.1)	472 (17.6)	3112 (28.2)	-	-	2911 (26.3)	352 (17.4)	2559 (28.3)	-
>10 million	-	1041 (7.6)	122 (4.5)	919 (8.3)	-	-	822 (7.4)	89 (4.4)	733 (8.1)	-
Unknown	-	3715 (27.1)	1002 (37.3)	2713 (24.6)	-	-	2939 (26.6)	746 (36.9)	2193 (24.3)	-
**Alcohol Consumption**	2.20	-	-	-	<0.001	2.31	-	-	-	<0.001
Non-drinker	-	8233 (60.0)	1778 (66.2)	6455 (58.4)	-	-	6571 (59.4)	1334 (66.0)	5237 (58.0)	-
Drinker	-	5497 (40.0)	907 (33.8)	4590 (41.6)	-	-	4485 (40.6)	688 (34.0)	3797 (42.0)	-
**Smoking status**	1.42				0.002	1.46	-	-	-	0.11
No-smokers	-	12,474 (90.9)	2398 (89.3)	10,076 (91.2)	-	-	10,000 (90.4)	1806 (89.3)	8194 (90.7)	-
Smokers	-	1256 (9.1)	287 (10.7)	969 (8.8)	-	-	1056 (9.6)	216 (10.7)	840 (9.3)	-
**Mental health status**	2.12				<0.001	**-**	-	-	-	-
No	-	11,320 (82.4)	2110 (78.6)	9210 (83.4)	-	-	-	-	-	-
Other		1543 (11.2)	331 (12.3)	1212 (11.0)	-	-	-	-	-	-
Yes	-	867 (6.3)	244(9.1)	623 (5.6)	-	-	-	-	-	-

**Table 2:** Multivariable and multivariate multinomial regression analyses for cancer screening behavior (breast cancer status is based on previously reported data) [11].

	*Cervical Cancer Screening Status*						*Breast Cancer Screening Status*					
	Screened Group (n = 6079) vs. Non-Screened Group			Intending to Screen Group (n = 2768) vs. Non-Screened Group			Screened Group (n = 5023) vs. Non-Screened Group			Intending to Screen Group (n = 2450) vs. Non-Screened Group		
	Adjusted Odds Ratio	95% CI	*p*-Values	Adjusted Odds Ratio	95% CI	*p*-Values	Adjusted Odds Ratio	95% CI	*p*-Values	Adjusted Odds Ratio	95% CI	*p*-Values
**Sexual minority women**												
No	Reference			Reference			Reference			Reference		
Yes	0.75	0.68–0.83	<0.001	0.63	0.56–0.71	<0.001	0.82	0.73–0.91	<0.001	0.59	0.52–0.68	<0.001
**Insurance Status**												
National Health Insurance	Reference			Reference			Reference			Reference		
Employee’s Health Insurance	1.95	1.79–2.11	<0.001	1.62	1.47–1.79	<0.001	1.61	1.47–1.76	<0.001	1.43	1.28–1.60	<0.001
Other	0.69	0.55–0.88	0.003	0.97	0.75–1.26	0.83	0.70	0.51–0.96	0.03	0.77	0.52–1.14	0.19
Uninsured	0.45	0.28–0.73	0.001	0.68	0.41–1.14	0.15	0.36	0.19–0.70	0.003	0.68	0.34–1.36	0.28
**Marital Status**												
Unmarried	Reference			Reference			Reference			Reference		
Married	1.56	1.44–1.7	<0.001	1.16	1.05–1.28	0.003	1.20	1.09–1.32	<0.001	1.30	1.16–1.46	<0.001
**Annual Household Income (JPY)**												
<5 million	Reference			Reference			Reference			Reference		
5–10 million	1.56	1.4–1.73	<0.001	1.34	1.18–1.52	<0.001	1.34	1.19–1.51	<0.001	1.30	1.13–1.49	<0.001
>10 million	1.90	1.61–2.24	<0.001	1.31	1.06–1.61	0.011	1.94	1.60–2.35	<0.001	1.25	0.99–1.58	0.06
Unknown	1.03	0.93–1.13	0.58	1.00	0.89–1.12	0.97	1.11	0.99–1.23	0.06	0.91	0.80–1.04	0.16
**Alcohol Consumption**												
Non-drinker	Reference			Reference			Reference			Reference		
Drinker	1.18	1.09–1.28	<0.001	1.16	1.06–1.28	0.002	1.25	1.15–1.37	<0.001	1.18	1.06–1.31	0.003
**Smoking Status**												
No-smoker	Reference			Reference			Reference			Reference		
Smoker	0.89	0.77–1.02	0.08	1.07	0.91–1.25	0.42	0.76	0.65–0.88	<0.001	1.09	0.92–1.29	0.31
**Mental Health Status**												
No	Reference			Reference			-			-		
Other	1.18	1.04–1.34	0.01	1.30	1.12–1.51	0.001	-	-	-	-	-	-
Yes	1.04	0.88–1.22	0.67	1.39	1.15–1.67	0.001	-	-	-	-	-	-

**Table 3:** Multinomial logistic regression analysis of cervical cancer screening status in subgroups of sexual minority women.

	*Cervical Cancer Screening Status*												
*Bisexual*	Screened Group (n = 76) vs. Non-Screened Group			Intending to Screen Group (n = 36) vs. Non-Screened Group			*Other*	Screened Group (n = 202) vs. Non-Screened Group			Intending to Screen Group (n = 85) vs. Non-Screened Group		
	Adjusted Odds Ratio	95% CI		Adjusted Odds Ratio	95% CI	*p*-Values		Adjusted Odds Ratio	95% CI	*p*-Values	Adjusted Odds Ratio	95% CI	*p*-Values
**Insurance Status**							**Insurance Status**						
National Health Insurance	Reference			Reference			National Health Insurance	Reference			Reference		
Employee’s Health Insurance	0.42	0.19–0.94	0.03	1.20	0.44–3.27	0.73	Employee’s Health Insurance	2.07	1.33–3.21	0.001	2.33	1.32–4.10	0.003
Other	0.46	0.09–2.19	0.33	1.99	0.37–10.59	0.42	Other	0.26	0.05–1.23	0.09	1.55	0.48–4.95	0.46
Uninsured	-	-	-	1.71	0.08–35.26	0.73	Uninsured	0.58	0.06–5.88	0.65	-	-	-
**Marital Status**							**Marital Status**						
Unmarried	Reference			Reference			Unmarried	Reference			Reference		
Married	2.85	1.23–6.57	0.01	1.67	0.62–4.53	0.31	Married	1.83	1.19–2.82	0.006	1.18	0.68–2.04	0.55
**Annual Household Income (JPY)**							**Annual Household Income (JPY)**						
<5 million	Reference			Reference			<5 million	Reference			Reference		
5–10 million	0.94	0.39–2.27	0.89	0.37	0.12–1.20	0.10	5–10 million	1.67	0.95–2.92	0.07	1.19	0.56–2.53	0.66
>10 million	0.40	0.10–1.56	0.19	0.15	0.02–1.45	0.10	>10 million	1.22	0.43–3.46	0.71	1.28	0.36–4.53	0.70
Unknown	0.74	0.23–2.39	0.61	1.42	0.41–4.97	0.58	Unknown	1.07	0.65–1.76	0.78	1.48	0.81–2.7	0.21
**Alcohol Consumption**							**Alcohol Consumption**						
Non-drinker	Reference			Reference			Non-drinker	Reference			Reference		
Drinker	1.91	0.90–4.04	0.09	1.39	0.57–3.41	0.47	Drinker	0.99	0.64–1.52	0.97	0.91	0.52–1.58	0.74
**Smoking status**							**Smoking status**						
No-smoker	Reference			Reference			No-smokers	Reference			Reference		
Smoker	0.93	0.35–2.44	0.88	1.29	0.43–3.89	0.65	Smokers	1.57	0.75–3.28	0.23	0.92	0.32–2.66	0.87
**Mental Health Status**							**Mental Health Status**						
No	Reference			Reference			No	Reference			Reference		
Other	0.31	0.11–0.82	0.02	1.17	0.42–3.24	0.77	Other	0.90	0.50–1.64	0.73	0.42	0.16–1.07	0.07
Yes	0.87	0.34–2.22	0.77	0.53	0.15–1.85	0.32	Yes	0.73	0.31–1.69	0.46	0.92	0.36–2.34	0.86
***Undecided***	**Screened Group (n = 257) vs. Non-Screened Group**			**Intending to Screen Group (n = 141) vs. Non-Screened Group**			***Unsure***	**Screened Group (n = 490) vs. Non-Screened Group**			**Intending to Screen Group (n = 171) vs. Non-Screened Group**		
	**Adjusted Odds Ratio**	**95% CI**	***p*-Values**	**Adjusted Odds Ratio**	**95% CI**	***p*-Values**		**Adjusted Odds Ratio**	**95% CI**	***p*-Values**	**Adjusted Odds Ratio**	**95% CI**	***p*-Values**
**Insurance Status**							**Insurance Status**						
National Health Insurance	Reference			Reference			National Health Insurance	Reference			Reference		
Employee’s Health Insurance	1.22	0.86–1.74	0.26	1.26	0.82–1.94	0.28	Employee’s Health Insurance	1.27	0.98–1.65	0.08	1.52	1.05–2.20	0.03
Other	0.92	0.44–1.94	0.83	1.23	0.54–2.80	0.62	Other	0.43	0.25–0.74	0.002	0.78	0.39–1.53	0.46
Uninsured	0.19	0.04–0.88	0.03	0.18	0.02–1.42	0.10	Uninsured	0.23	0.05–1.04	0.06	0.68	0.15–3.14	0.63
**Marital Status**							**Marital Status**						
Unmarried	Reference			Reference			Unmarried	Reference			Reference		
Married	1.65	1.17–2.34	0.005	1.29	0.85–1.97	0.23	Married	1.94	1.50–2.50	<0.001	1.30	0.91–1.86	0.15
**Annual Household Income (JPY)**							**Annual Household Income (JPY)**						
<5 million	Reference			Reference			<5 million	Reference			Reference		
5–10 million	1.98	1.23–3.21	0.005	1.64	0.92–2.93	0.09	5–10 million	1.11	0.75–1.66	0.6	1.03	0.59–1.82	0.91
>10 million	0.84	0.38–1.84	0.66	0.49	0.16–1.52	0.22	>10 million	1.53	0.78–2.98	0.22	1.21	0.47–3.13	0.69
Unknown	1.01	0.69–1.49	0.95	1.02	0.64–1.61	0.94	Unknown	0.90	0.68–1.18	0.44	0.97	0.66–1.44	0.90
**Alcohol Consumption**							**Alcohol Consumption**						
Non-drinker	Reference			Reference			Non-drinker	Reference			Reference		
Drinker	1.26	0.87–1.81	0.22	1.33	0.87–2.06	0.19	Drinker	1.28	0.98–1.68	0.07	1.40	0.97–2.02	0.07
**Smoking Status**							**Smoking Status**						
No-smokers	Reference			Reference			No-smokers	Reference			Reference		
Smokers	0.81	0.47–1.41	0.46	0.72	0.37–1.41	0.34	Smokers	0.83	0.54–1.28	0.41	1.51	0.91–2.51	0.11
**Mental Health Status**							**Mental Health Status**						
No	Reference			Reference			No	Reference			Reference		
Other	1.32	0.78–2.22	0.30	1.64	0.90–2.98	0.11	Other	1.11	0.74–1.67	0.62	0.93	0.52–1.68	0.81
Yes	1.95	1.09–3.51	0.03	2.53	1.32–4.85	0.005	Yes	0.96	0.57–1.60	0.87	1.50	0.82–2.73	0.19

**Table 4:** Multivariable and multivariate multinomial regression analyses for cancer screening behavior in the population of sexual minority women (breast cancer status is based on previously reported data) [11].

	*Cervical Cancer Screening Status*						*Breast Cancer Screening Status*					
	Screened Group (n = 1039) vs. Non-Screened Group			Intending to Screen Group (n = 443) vs. Non-Screened Group			Screened Group (n = 878) vs. Non-Screened Group			Intending to Screen Group (n = 331) vs. Non-Screened Group		
	Adjusted Odds Ratio	95% CI	*p*-Value	Adjusted Odds Ratio	95% CI	*p*-Value	Adjusted Odds Ratio	95% CI	*p*-Value	Adjusted Odds Ratio	95% CI	*p*-Value
**Insurance Status**												
National Health Insurance	Reference			Reference			Reference			Reference		
Employee’s Health Insurance	1.28	1.07–1.53	0.01	1.54	1.22–1.94	<0.001	1.05	0.86–1.29	0.62	1.27	0.97–1.67	0.08
Other	0.53	0.36–0.78	0.002	1.02	0.65–1.58	0.95	0.46	0.27–0.77	0.003	0.53	0.25–1.11	0.09
Uninsured	0.21	0.08–0.55	0.001	0.42	0.14–1.20	0.10	0.23	0.08–0.70	0.01	0.17	0.02–1.29	0.09
**Marital Status**												
Unmarried	Reference			Reference			Reference			Reference		
Married	1.84	1.54–2.20	<0.001	1.27	1.01–1.59	0.04	1.48	1.21–1.81	<0.001	1.33	1.01–1.74	0.04
**Annual Household Income (JPY)**												
<5 million	Reference			Reference			Reference			Reference		
5–10 million	1.43	1.12–1.84	0.01	1.11	0.80–1.54	0.55	1.27	0.95–1.70	0.10	1.25	0.85–1.82	0.25
>10 million	1.13	0.75–1.72	0.56	0.80	0.45–1.44	0.46	1.19	0.72–1.94	0.50	1.00	0.51–1.96	1.00
Unknown	0.94	0.77–1.14	0.53	0.96	0.75–1.23	0.74	1.08	0.86–1.34	0.51	1.02	0.76–1.38	0.87
**Alcohol Consumption**												
Non-drinker	Reference			Reference			Reference			Reference		
Drinker	1.26	1.05–1.52	0.01	1.30	1.03–1.64	0.03	1.39	1.13–1.71	0.002	1.13	0.85–1.49	0.40
**Smoking Status**												
No-smoker	Reference			Reference			Reference			Reference		
Smoker	0.97	0.73–1.29	0.84	1.13	0.80–1.60	0.48	0.84	0.61–1.15	0.28	1.11	0.74–1.65	0.62
**Mental Health Status**												
No	Reference			Reference			Reference			Reference		
Other	1.03	0.79–1.34	0.81	1.11	0.80–1.55	0.54	-	-	-	-	-	-
Yes	1.27	0.93–1.73	0.14	1.55	1.07–2.24	0.02	-	-	-	-	-	-

**Table 5:** Multivariable multinomial regression analysis of cancer screening behavior among sexual minority and women who are not part of a sexual minority: a comparative assessment of breast-only, cervical-only, and non-participation groups relative to complete screening.

	Participants in Breast Cancer Screening (n = 681) vs. Participants in All Screenings (n = 5465)			Participants in Cervical Cancer Screening (n = 226) vs. Participants in All Screenings (n = 5465)			Non-Participants in Any Screening (2561) vs. Participants in All Screenings (n = 5465)		
	Adjusted Odds Ratio	95% CI	*p*-Values	Adjusted Odds Ratio	95% CI	*p*-Values	Adjusted Odds Ratio	95% CI	*p*-Values
**Sexual Minority Women**									
No	Reference			Reference			Reference		
Yes	0.95	0.76–1.18	0.62	1.23	0.88–1.72	0.22	1.29	1.14–1.45	<0.001
**Insurance Status**									
National Health Insurance	Reference			Reference			Reference		
Employee’s Health Insurance	0.45	0.38–0.54	<0.001	0.70	0.53–0.92	0.01	0.48	0.43–0.53	<0.001
Other	0.61	0.34–1.10	0.10	1.10	0.50–2.43	0.82	1.50	1.14–1.96	0.003
**Marital Status**									
Unmarried	Reference			Reference			Reference		
Married	0.78	0.65–0.92	0.004	1.04	0.77–1.40	0.80	0.81	0.73–0.90	<0.001
**Annual Household Income (JPY)**									
<5 million	Reference			Reference			Reference		
5–10 million	0.68	0.54–0.86	0.001	1.07	0.75–1.53	0.72	0.68	0.59–0.78	<0.001
>10 million	0.52	0.35–0.78	0.002	0.81	0.45–1.46	0.48	0.52	0.41–0.65	<0.001
Unknown	0.78	0.64–0.95	0.01	1.02	0.73–1.42	0.91	0.88	0.78–0.98	0.03
**Alcohol Consumption**									
Non-drinker	Reference			Reference			Reference		
Drinker	0.88	0.74–1.03	0.12	0.77	0.58–1.02	0.07	0.87	0.79–0.96	0.007
**Smoking Status**									
No-smoker	Reference			Reference			Reference		
Smoker	0.72	0.53–0.98	0.03	1.58	1.08–2.32	0.02	1.09	0.93–1.28	0.27
**Mental Health Status**									
No	Reference			Reference			Reference		
Other	1.00	0.77–1.29	0.97	1.22	0.81–1.84	0.34	0.82	0.69–0.97	0.02
Yes	0.75	0.51–1.12	0.16	1.70	1.06–2.74	0.03	0.92	0.75–1.14	0.46

## Data Availability

Data generated during the study can be obtained from the corresponding author upon reasonable request.

## Supplementary Materials

The following supporting information can be downloaded at: https://www.mdpi.com/article/10.3390/cancers17091411/s1, Table S1: Univariable Multinomial Logistic Regression Results; Table S2: Detailed Breakdown of Demographic Characteristics and Screening Behaviors within sexual minority women subgroups; Table S3: Multivariable and Multivariate Multinomial Regression Analyses for Cervical Cancer Screening Behavior (Sexual Minority Women Excluding “Unsure”); Table S4: Multivariable and Multivariate Multinomial Regression Analyses for Cervical Cancer Screening Behavior (Sexual Minority Women With “Unsure” as Separate Category); Table S5: Weighted Multinomial Logistic Regression Analysis of Cervical Cancer Screening Status Among Sexual Minority Women; Table S6: Contingency Table of Cervical and Breast Cancer Screening Participation and χ^2^ Test by Sexual minority women identity (Restricted to Participants Aged 40 and Above).
