# Supplementary material for: Disparities in Cervical and Breast Cancer Screening Among Sexual Minority Women in Japan: A Comparative Cross-Sectional Study
Source: Cancers (Basel). 2025 Apr 23;17(9):1411. doi: 10.3390/cancers17091411 (PMC12071106; doi:10.3390/cancers17091411)
Supplement: Supplementary file 1 [file cancers-17-01411-s001.zip › cancers-3577625-supplementary.pdf]

Supplementary Table S1. Univariable Multinomial Logistic Regression Results.

Variable	LRT p-value
**Sexual minority women identity**	**<0.001**
**Insurance Status**	**<0.001**
**Marital Status**	**<0.001**
**Annual Household Income (JPY)**	**<0.001**
**Alcohol Consumption**	**<0.001**
**Smoking status**	**0.005**
**Mental health status**	**<0.001**

Supplementary Table S2. Detailed Breakdown of Demographic Characteristics and Screening Behaviors within sexual minority women subgroups.

	Total n (%)	Homosexual n (%)	Bisexual n (%)	Other n (%	Undecided n (%)	Unsure n (%)
**Overall**	2685 (100.0%)	45 (1.7%)	174 (6.5%)	489 (18.2%)	731 (27.2%)	1246 (46.4%)
**Age**						
20s	663 (24.7%)	20 (44.4%)	89 (51.1%)	61 (12.5%)	232 (31.7%)	267 (20.9%)
30s	378 (14.1%)	10 (22.2%)	36 (20.7%)	47 (9.6%)	115 (15.7%)	170 (13.6%)
40s	396 (14.7%)	5 (11.1%)	24 (13.8%)	57 (11.7%)	111 (15.2%)	199 (15.9%)
50s	347 (12.9%)	3 (6.7%)	11 (6.3%)	73 (14.9%)	75 (10.3%)	185 (14.9%)
60s	397 (14.8%)	6 (13.3%)	5 (2.9%)	115 (23.5%)	78 (10.7%)	193 (15.5%)
70s	438 (16.3%)	0 (0.0%)	9 (5.2%)	126 (25.8%)	100 (13.7%)	203 (16.3%)
80s	66 (2.5%)	1 (2.2%)	0 (0.0%)	10 (2.0%)	20 (2.7%)	35 (2.8%)
**Cervical Cancer Screening**						
Screened	1039 (38.7%)	14 (31.1%)	76 (43.7%)	202 (41.3%)	257 (35.2%)	490 (39.3%)
└─ No abnormality	943 (90.8%)	11 (78.6%)	65 (85.5%)	187 (92.6%)	228 (88.7%)	452 (92.2%)
└─ Abnormality	52 (5.0%)	0 (0.0%)	5 (6.6%)	9 (4.5%)	14 (5.4%)	24 (4.9%)
└─ Unknown result	44 (4.2%)	3 (21.4%)	6 (7.9%)	6 (3.0%)	15 (5.8%)	14 (2.9%)
Planned Screening	443 (16.5%)	10 (22.2%)	36 (20.7%)	85 (17.4%)	141 (19.3%)	171 (13.7%)
No Screening	1203 (44.8%)	21 (46.7%)	62 (35.6%)	202 (41.3%)	333 (45.5%)	585 (47.0%)
**Insurance Status**						
National Health Insurance	1240 (46.2%)	13 (28.9%)	64 (36.8%)	262 (53.6%)	325 (44.5%)	576 (46.2%)
Employee's Health Insurance	1232 (45.9%)	31 (68.9%)	96 (55.2%)	205 (41.9%)	344 (47.1%)	556 (44.6%)
Other	172 (6.4%)	0 (0.0%)	12 (6.9%)	18 (3.7%)	45 (6.2%)	97 (7.8%)
Uninsured	41 (1.5%)	1 (2.2%)	2 (1.1%)	4 (0.8%)	17 (2.3%)	17 (1.4%)
**Marital Status**						
Unmarried	1356 (50.5%)	32 (71.1%)	110 (63.2%)	211 (43.1%)	395 (54.0%)	608 (48.8%)
Married	1329 (49.5%)	13 (28.9%)	64 (36.8%)	278 (56.9%)	336 (46.0%)	638 (51.2%)
**Annual Household Income (JPY)**						
<5 million	1089 (40.6%)	28 (62.2%)	85 (48.9%)	218 (44.6%)	327 (44.7%)	431 (34.6%)
5-10 million	472 (17.6%)	10 (22.2%)	51 (29.3%)	104 (21.3%)	133 (18.2%)	174 (14.0%)
>10 million	122 (4.5%)	1 (2.2%)	14 (8.0%)	23 (4.7%)	34 (4.7%)	50 (4.0%)
Unknown	1002 (37.3%)	6 (13.3%)	24 (13.8%)	144 (29.4%)	237 (32.4%)	591 (47.4%)
**Alcohol Consumption**						
Non-drinker	1778 (66.2%)	26 (57.8%)	96 (55.2%)	308 (63.0%)	497 (68.0%)	851 (68.3%)
Drinker	907 (33.8%)	19 (42.2%)	78 (44.8%)	181 (37.0%)	234 (32.0%)	395 (31.7%)
**Smoking status**						
No-smokers	2398 (89.3%)	36 (80.0%)	143 (82.2%)	449 (91.8%)	650 (88.9%)	1120 (89.9%)
Smokers	287 (10.7%)	9 (20.0%)	31 (17.8%)	40 (8.2%)	81 (11.1%)	126 (10.1%)
**Mental health status**						
No	2110 (78.6%)	27 (60.0%)	103 (59.2%)	389 (79.6%)	564 (77.2%)	1027 (82.4%)
Other	331 (12.3%)	12 (26.7%)	37 (21.3%)	63 (12.9%)	91 (12.4%)	128 (10.3%)
Yes	244 (9.1%)	6 (13.3%)	34 (19.5%)	37 (7.6%)	76 (10.4%)	91 (7.3%)

Supplementary Table S3. Multivariable and Multivariate Multinomial Regression Analyses for Cervical Cancer Screening Behavior (Sexual Minority Women Excluding "Unsure").

	Screened Group (n=5,589) vs. Non-screened Group			Intending to Screen Group (n=2,597) vs. Non-screened Group		
	**Adjusted Odds Ratio**	**95% CI**	**p-values**	**Adjusted Odds Ratio**	**95% CI**	**p-values**
**Sexual minority women identity**						
No	Reference			Reference		
Yes	0.76	0.67 - 0.86	<0.001	0.73	0.63 - 0.86	<0.001
**Insurance Status**						
National Health Insurance	Reference			Reference		
Employee's Health Insurance	2.04	1.87 - 2.22	<0.001	1.65	1.48 - 1.82	<0.001
Other	0.77	0.59 - 1.01	0.06	1.05	0.79 - 1.39	0.76
Uninsured	0.49	0.29 - 0.82	0.007	0.69	0.40 - 1.19	0.18
**Marital Status**						
Unmarried	Reference			Reference		
Married	1.53	1.40 - 1.67	<0.001	1.15	1.04 - 1.27	0.01
**Annual Household Income (JPY)**						
<5 million	Reference			Reference		
5-10 million	1.60	1.43 - 1.78	<0.001	1.37	1.20 - 1.56	<0.001
>10 million	1.93	1.63 - 2.29	<0.001	1.32	1.07 - 1.63	0.01
Unknown	1.05	0.94 - 1.16	0.41	1.02	0.90 - 1.15	0.78
**Alcohol Consumption**						
Non-drinker	Reference			Reference		
Drinker	1.17	1.07 - 1.27	<0.001	1.14	1.03 - 1.27	0.01
**Smoking status**						
No-smokers	Reference			Reference		
Smokers	0.89	0.77 - 1.03	0.12	1.04	0.88 - 1.22	0.68
**Mental health status**						
No	Reference			Reference		
Other	1.19	1.04 - 1.36	0.01	1.32	1.13 - 1.54	<0.001
Yes	1.05	0.88 - 1.25	0.58	1.37	1.12 - 1.66	0.002

Supplementary Table S4. Multivariable and Multivariate Multinomial Regression Analyses for Cervical Cancer Screening Behavior (Sexual Minority Women With "Unsure" as Separate Category).

	Screened Group (n=6,079) vs. Non-screened Group			Intending to Screen Group (n=2,768) vs. Non-screened Group		
	**Adjusted Odds Ratio**	**95% CI**	**p-values**	**Adjusted Odds Ratio**	**95% CI**	**p-values**
**Sexual minority women identity**						
No	Reference			Reference		
Yes	0.76	0.67 - 0.86	<0.001	0.74	0.63 - 0.86	<0.001
Unsure	0.75	0.66 - 0.86	<0.001	0.51	0.43 - 0.62	<0.001
**Insurance Status**						
National Health Insurance	Reference			Reference		
Employee's Health Insurance	1.95	1.79 - 2.11	<0.001	1.62	1.47 - 1.79	<0.001
Other	0.70	0.55 - 0.88	0.003	0.98	0.76 - 1.28	0.89
Uninsured	0.45	0.28 - 0.73	0.001	0.68	0.41 - 1.14	0.14
**Marital Status**						
Unmarried	Reference			Reference		
Married	1.56	1.44 - 1.70	<0.001	1.17	1.06 - 1.29	0.003
**Annual Household Income (JPY)**						
<5 million	Reference			Reference		
5-10 million	1.56	1.40 - 1.73	<0.001	1.34	1.18 - 1.52	<0.001
>10 million	1.90	1.61 - 2.24	<0.001	1.31	1.07 - 1.61	0.01
Unknown	1.03	0.93 - 1.13	0.56	1.01	0.90 - 1.14	0.83
**Alcohol Consumption**						
Non-drinker	Reference			Reference		
Drinker	1.18	1.09 - 1.28	<0.001	1.16	1.05 - 1.28	0.002
**Smoking status**						
No-smokers	Reference			Reference		
Smokers	0.89	0.77 - 1.02	0.08	1.07	0.91 - 1.25	0.42
**Mental health status**						
No	Reference			Reference		
Other	1.18	1.04 - 1.34	0.01	1.29	1.11 - 1.50	0.001
Yes	1.04	0.88 - 1.22	0.68	1.38	1.14 - 1.66	0.001

Supplementary Table S5. Weighted Multinomial Logistic Regression Analysis of Cervical Cancer Screening Status Among Sexual Minority Women.

	Cervical Cancer Screening Status					
	**Screened Group (n=6,079) vs. Non-screened Group**			**Intending to Screen Group (n=2,768) vs. Non-screened Group**		
	Adjusted Odds Ratio	95% CI	p-values	Adjusted Odds Ratio	95% CI	p-values
**Sexual minority women identity**						
No	Reference			Reference		
Yes	0.75	0.66 - 0.86	<0.001	0.63	0.54 - 0.74	<0.001
**Insurance Status**						
National Health Insurance	Reference			Reference		
Employee's Health Insurance	2.05	1.89 - 2.23	<0.001	1.65	1.49 - 1.82	<0.001
Other	0.73	0.57 - 0.94	0.01	0.97	0.73 - 1.27	0.81
Uninsured	0.52	0.31 - 0.85	0.01	0.75	0.45 - 1.28	0.30
**Marital Status**						
Unmarried	Reference			Reference		
Married	1.53	1.41 - 1.66	<0.001	1.15	1.04 - 1.27	0.006
**Annual Household Income (JPY)**						
<5 million	Reference			Reference		
5-10 million	1.57	1.41 - 1.74	<0.001	1.36	1.20 - 1.55	<0.001
>10 million	1.99	1.69 - 2.35	<0.001	1.37	1.12 - 1.68	0.002
Unknown	1.04	0.94 - 1.15	0.45	1.00	0.89 - 1.13	0.97
**Alcohol Consumption**						
Non-drinker	Reference			Reference		
Drinker	1.17	1.08 - 1.27	<0.001	1.15	1.05 - 1.27	0.004
**Smoking status**						
No-smokers	Reference			Reference		
Smokers	0.87	0.76 – 1.00	0.05	1.06	0.90 - 1.24	0.51
**Mental Health Status**						
No	Reference			Reference		
Other	1.21	1.06 - 1.37	0.004	1.33	1.14 - 1.54	<0.001
Yes	1.01	0.85 - 1.19	0.94	1.36	1.13 - 1.65	0.001

Supplementary Table S6. Contingency Table of Cervical and Breast Cancer Screening Participation and χ^2 Test* by Sexual minority women identity (Restricted to Participants Aged 40 and Above).

Group	Neither Participated	Breast Not & Cervical Participated	Breast Participated & Cervical Not Participated	Both Participated
**Overall**	2561 (28.7%)	226 (2.5%)	681 (7.6%)	5465 (61.2%)
**Sexual minority women**	576 (35.0%)	47 (2.9%)	116 (7.1%)	905 (55.0%)
**Women who are not part of a sexual minority**	1985 (27.2%)	179 (2.5%)	565 (7.8%)	4560 (62.6%)

*A significant difference was observed between sexual minority women and women who are not part of a sexual minority ($p < 0.001$).

Questionnaire Items and Analytical Categorization in the Study

Question	Answer	Categorization in Analysis
1. Have you undergone breast (e.g., mammography or breast ultrasound) / cervical cancer screening (e.g., cervical cytology) cancer screening in the past two years?	Received screening with no abnormal findings<br>Received screening with abnormal findings<br>Received screening, but results are unknown (e.g., results not yet available or not remembered)	Screened
	Not screened, but intending to screen in the future (due to reasons unrelated to COVID-19)<br>Not screened, but intending to screen in the future (due to COVID-19-related reasons)	Attempted but not screened
	Not screened, and not intending to screen in the future (due to reasons unrelated to COVID-19)<br>Not screened, and not intending to screen in the future (due to COVID-19-related reasons)	Not screened
2. Please select the option that best describes your sexual orientation.	Heterosexual (not gay or lesbian)	Non-LGBTQ
	Homosexual (gay or lesbian)<br>Bisexual<br>Other<br>Undecided<br>Unsure	LGBTQ
3. What is your current health insurance enrollment status?	National Health Insurance (municipal)<br>National Health Insurance (union)	National Health Insurance
	Employee's Health Insurance (Japan Health Insurance Association)<br>Employee's Health Insurance (Health Insurance Union)<br>Employee's Health Insurance (Mutual Aid Association)<br>Employee's Health Insurance (Seafarers' Insurance, other)	Employee's Health Insurance
	Public Assistance<br>Other	Other
	Uninsured (no health insurance or expired insurance)	Uninsured
4. Do you currently have a spouse?	Married (before March 2020)<br>Married (April 2020 - March 2023)<br>Married (after April 2023)	Married
	Never married<br>Widowed (before March 2020)<br>Widowed (April 2020 - March 2023)<br>Widowed (after April 2023)<br>Divorced (before March 2020)<br>Divorced (April 2020 - March 2023)	Unmarried

	Divorced (after April 2023)	
5. What is your approximate annual household income (before tax)?	0 JPY<br>< 0.5 million JPY<br>0.5-1 million JPY<br>1-2 million JPY<br>2-3 million JPY<br>3-4 million JPY<br>4-5 million JPY	<5 million JPY
	5-6 million JPY<br>6-7 million JPY<br>7-8 million JPY<br>8-9 million JPY<br>9-10 million JPY	5-10 million JPY
	10-12 million JPY<br>12-14 million JPY<br>14-16 million JPY<br>16-18 million JPY<br>18-20 million JPY<br>>2,0 million JPY	>10 million JPY
	Prefer not to answer<br>Do not know	Unknown
6. Do you currently consume alcohol?	Never used (drank)<br>Tried at least once but did not use regularly<br>Used to drink regularly but have stopped	Non-drinker
	Drink occasionally<br>Drink almost daily	Drinker
7. Do you currently smoke or use tobacco products?	Never smoked (used)<br>Tried at least once but did not use regularly<br>Used to smoke (use) regularly but have stopped	Non-smoker
	Smoke (use) occasionally<br>Smoke (use) almost daily	Smoker
8. Do you currently have depression or any other mental disorder?	Never had any mental disorder	None
	No current disorder but had in the past	Other
	Currently have a disorder (under medical treatment with medication)<br>Currently have a disorder (under medical treatment without medication)<br>Currently have a disorder (not receiving medical treatment)	Yes
